# Supplementary material for: Epimesatines P–S: Four Undescribed Flavonoids from Epimedium sagittatum Maxim. and Their Cytotoxicity Activities
Source: Molecules. 2024 Oct 4;29(19):4711. doi: 10.3390/molecules29194711 (PMC11477557; doi:10.3390/molecules29194711)
Supplement: Supplementary file 1 [file molecules-29-04711-s001.zip › molecules-3221289-supplementary.pdf]

Supplementary Files

Epimesatines P–S: Four Undescribed Flavonoids from *Epimedium sagittatum* Maxim. and Their Cytotoxicity Activities

Shuang-Shuang Xie [1,2], Xiang Yu [1,2], Jing-Ke Zhang [1,2], Zhi-You Hao [1,2], Xiao-Ke Zheng [1,2] and Wei-Sheng Feng [1,2,3,*]

1 School of Pharmacy, Henan University of Chinese Medicine, Zhengzhou 450046, China
2 The Engineering and Technology Center for Chinese Medicine Development of Henan Province, Zhengzhou 450046, China
3 Co-Construction Collaborative Innovation Center for Chinese Medicine and Respiratory Diseases by Henan & Education Ministry of China, Zhengzhou 450046, China
* Correspondence: fwsh@hactcm.edu.cn

List of Supplementary Materials

Figure S1. ^{1}H NMR (500 MHz) spectrum of **1** in Acetone-d_6

Figure S2. DEPT and ^{13}C NMR (125 MHz) spectra of **1** in Acetone-d_6

Figure S3. HSQC NMR spectrum of **1** in Acetone-d_6

Figure S4. HMBC NMR spectrum of **1** in Acetone-d_6

Figure S5. ^{1}H-^{1}H COSY NMR spectrum of **1** in Acetone-d_6

Figure S6. NOESY NMR spectrum of **1** in Acetone-d_6

Figure S7. IR spectrum of **1**

Figure S8. UV spectrum of **1**

Figure S9. HRESIMS spectrum of **1**

Figure S10. ^{1}H NMR (500 MHz) spectrum of **2** in Acetone-d_6

Figure S11. DEPT and ^{13}C NMR (125 MHz) spectra of **2** in Acetone-d_6

Figure S12. HSQC NMR spectrum of **2** in Acetone-d_6

Figure S13. HMBC NMR spectrum of **2** in Acetone-d_6

Figure S14. ^{1}H-^{1}H COSY NMR spectrum of **2** in Acetone-d_6

Figure S15. NOESY NMR spectrum of **2** in Acetone-d_6

Figure S16. IR spectrum of **2**

Figure S17. UV spectrum of **2**

Figure S18. HRESIMS spectrum of **2**

Figure S19. ^{1}H NMR (500 MHz) spectrum of **3** in Acetone-d_6

Figure S20. DEPT and ^{13}C NMR (125 MHz) spectra of **3** in Acetone-d_6

Figure S21. HSQC NMR spectrum of **3** in Acetone-d_6

Figure S22. HMBC NMR spectrum of **3** in Acetone-d_6

Figure S23. ^{1}H-^{1}H COSY NMR spectrum of **3** in Acetone-d_6

Figure S24. NOESY NMR spectrum of **3** in Acetone-d_6

Figure S25. IR spectrum of **3**

Figure S26. UV spectrum of **3**

Figure S27. HRESIMS spectrum of **3**

Figure S28. ^{1}H NMR (500 MHz) spectrum of **4** in Acetone-d_6

Figure S29. DEPT and ^{13}C NMR (125 MHz) spectra of **4** in Acetone-d_6

Figure S30. HSQC NMR spectrum of **4** in Acetone-d_6

Figure S31. HMBC NMR spectrum of **4** in Acetone-d_6

Figure S32. ^1H-^1H COSY NMR spectrum of **4** in Acetone-d_6

Figure S33. NOESY NMR spectrum of **4** in Acetone-d_6

Figure S34. IR spectrum of **4**

Figure S35. UV spectrum of **4**

Figure S36. HRESIMS spectrum of **4**

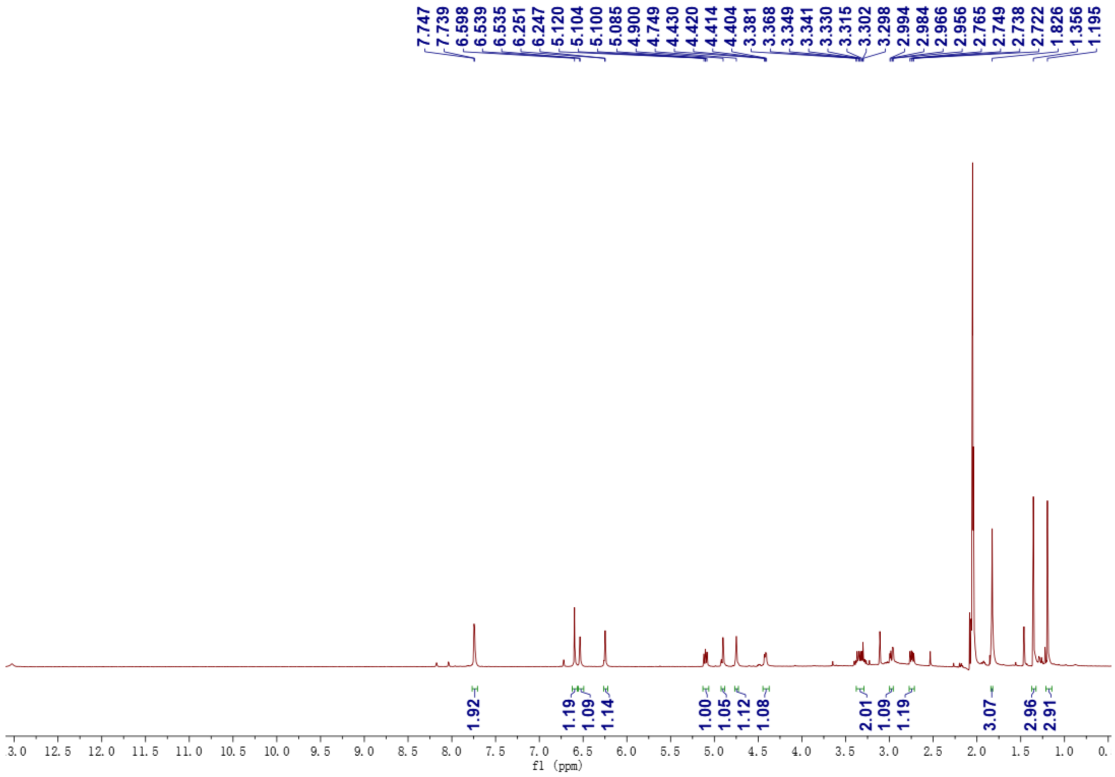

Figure S1. ^{1}H NMR (500 MHz) spectrum of **1** in Acetone-d_6.

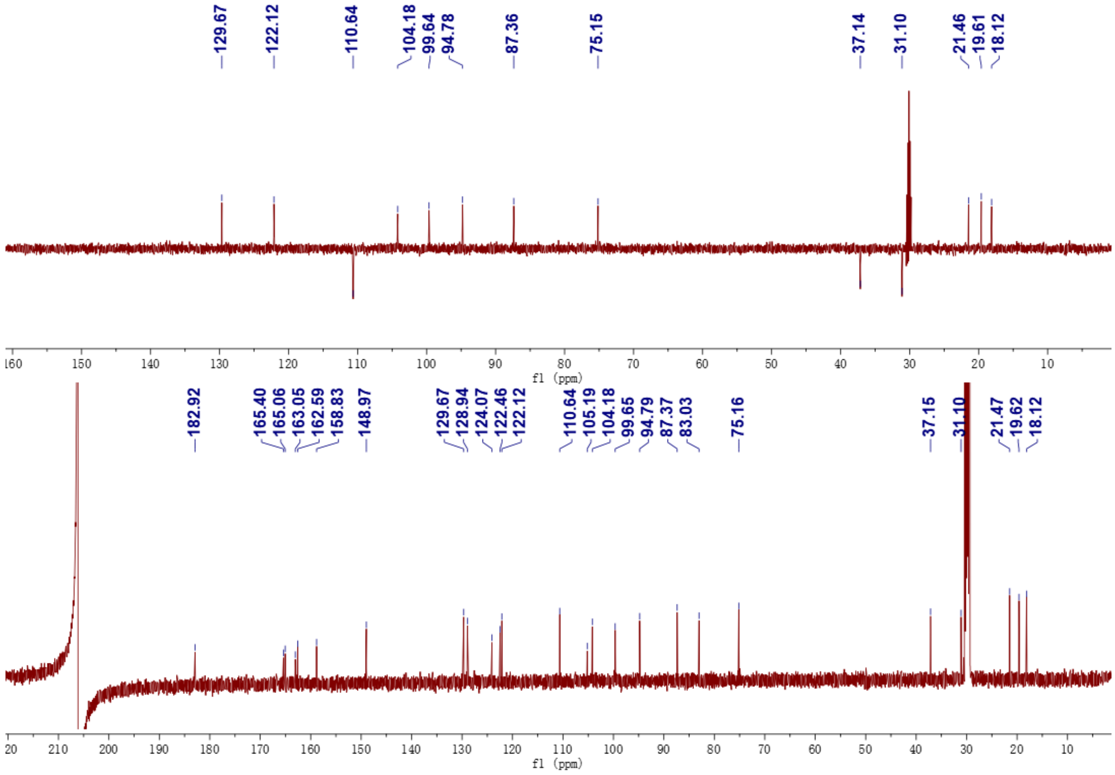

Figure S2. DEPT and ^{13}C NMR (125 MHz) spectra of **1** in Acetone-d_6.

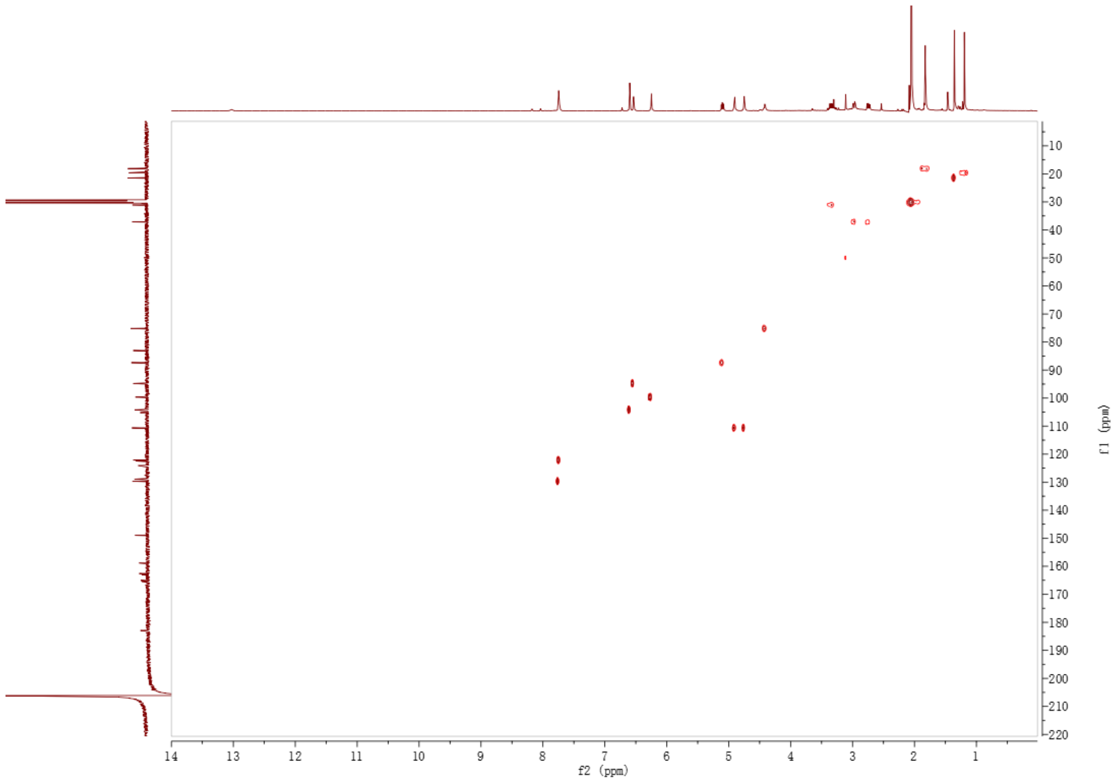

Figure S3. HSQC NMR spectrum of **1** in Acetone-d_6.

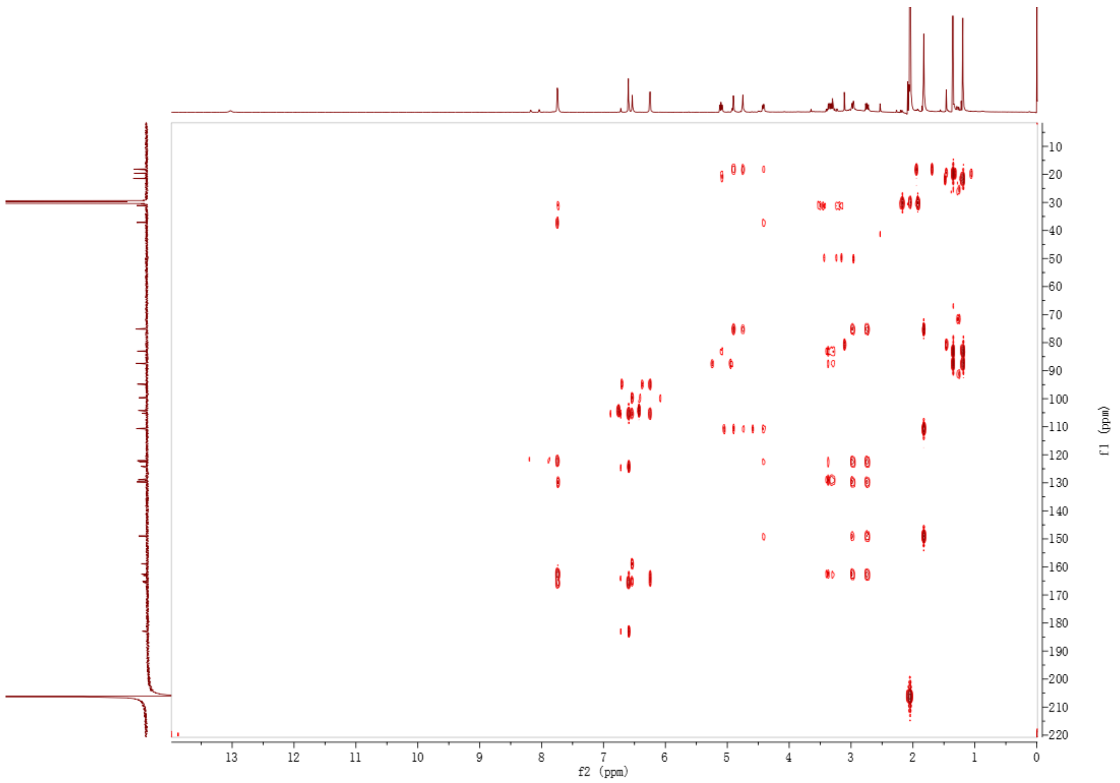

Figure S4. HMBC NMR spectrum of **1** in Acetone-d_6.

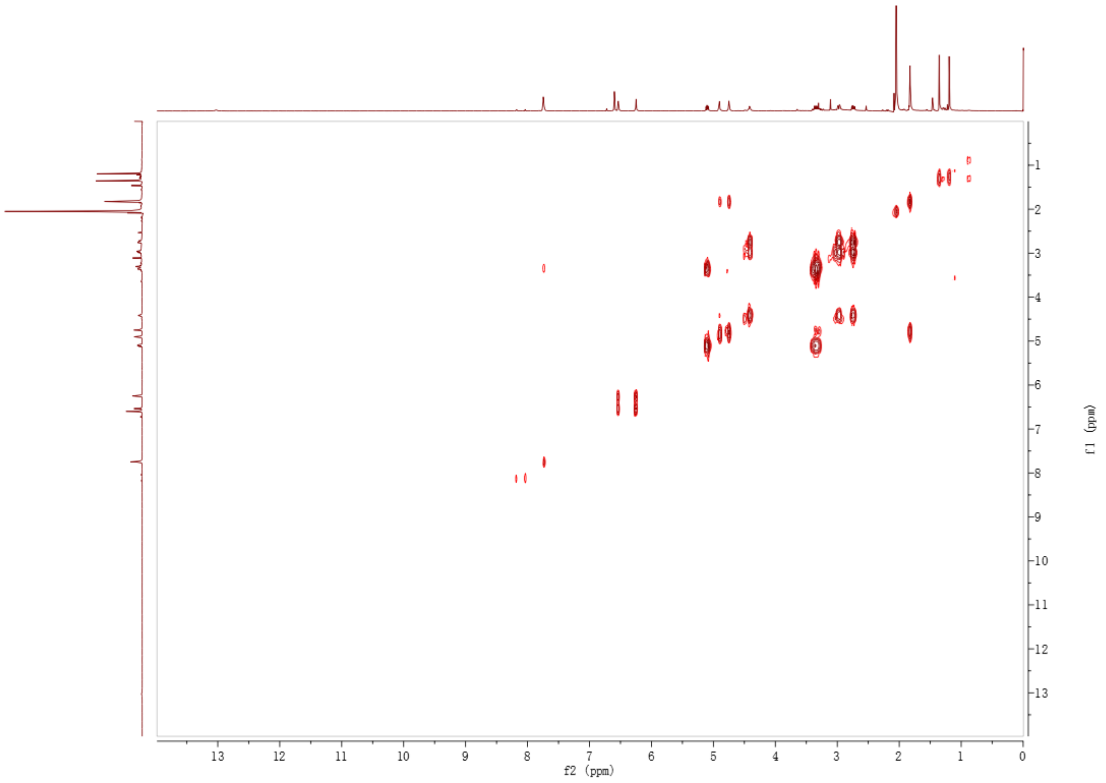

Figure S5. ^{1}H-^{1}H COSY NMR spectrum of **1** in Acetone-d_6.

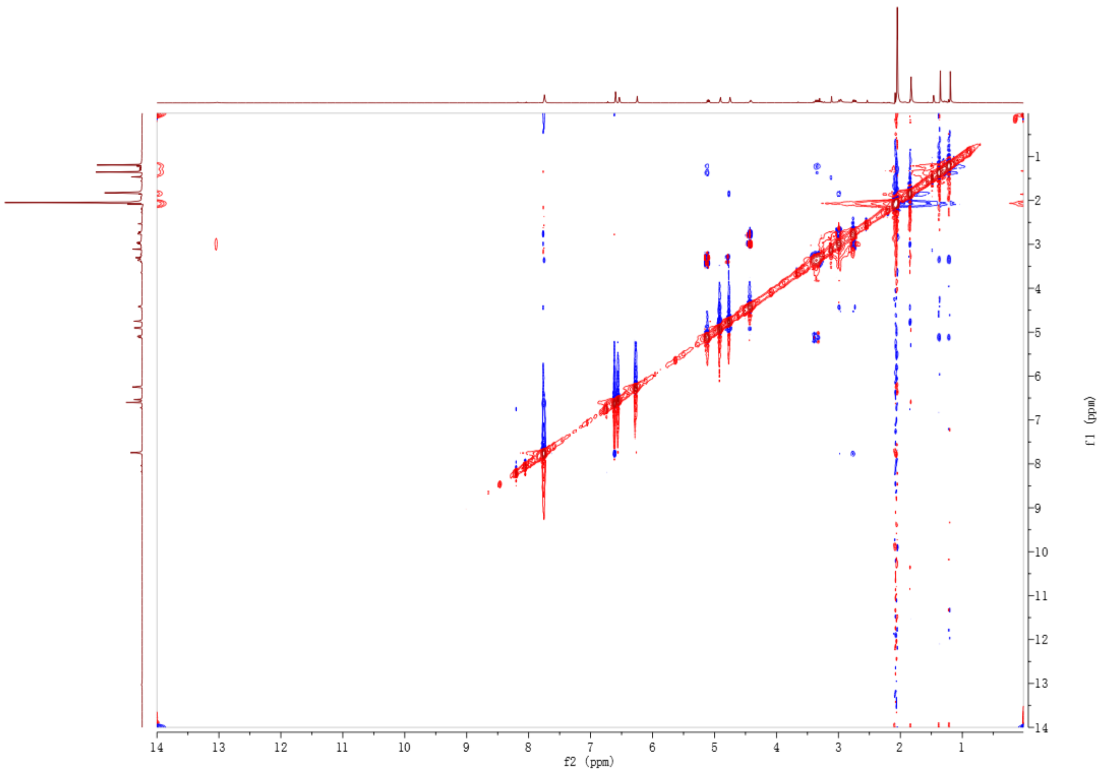

Figure S6. NOESY NMR spectrum of **1** in Acetone-d_6.

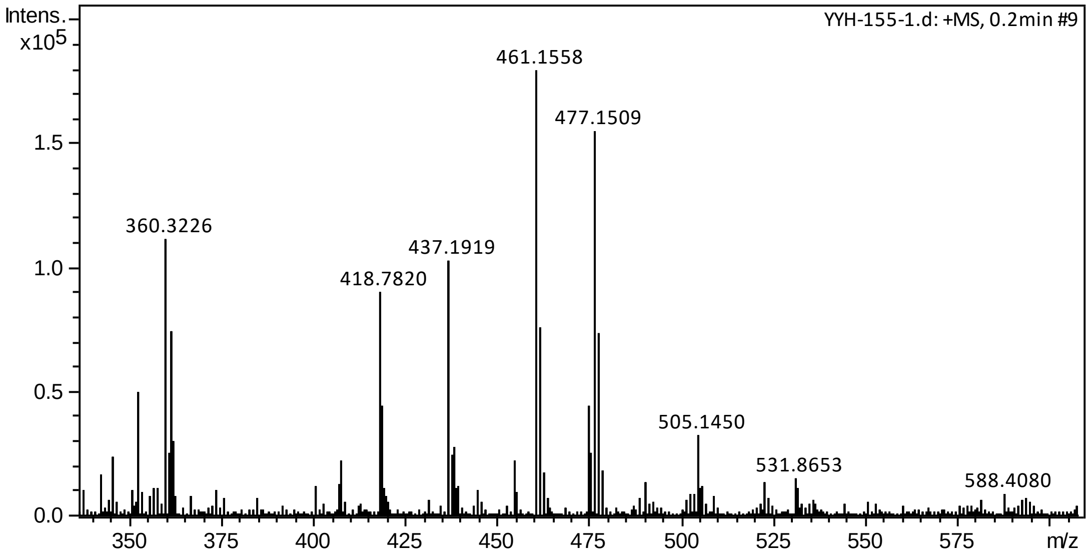

Figure S7. HRESIMS spectrum of **1**.

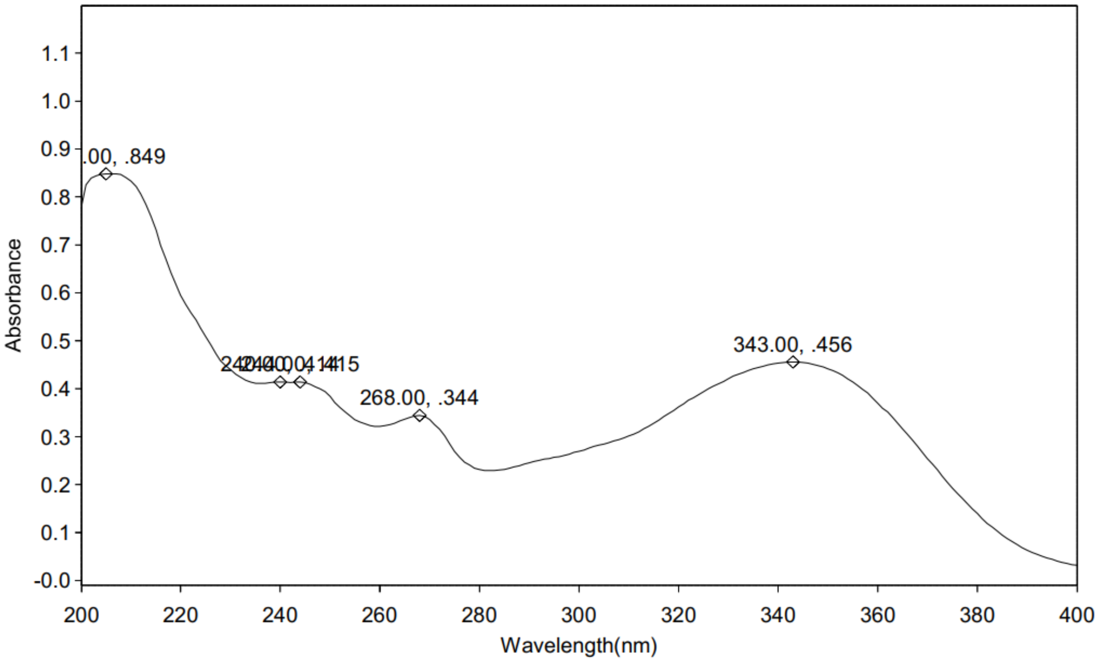

Figure S8. UV spectrum of **1**.

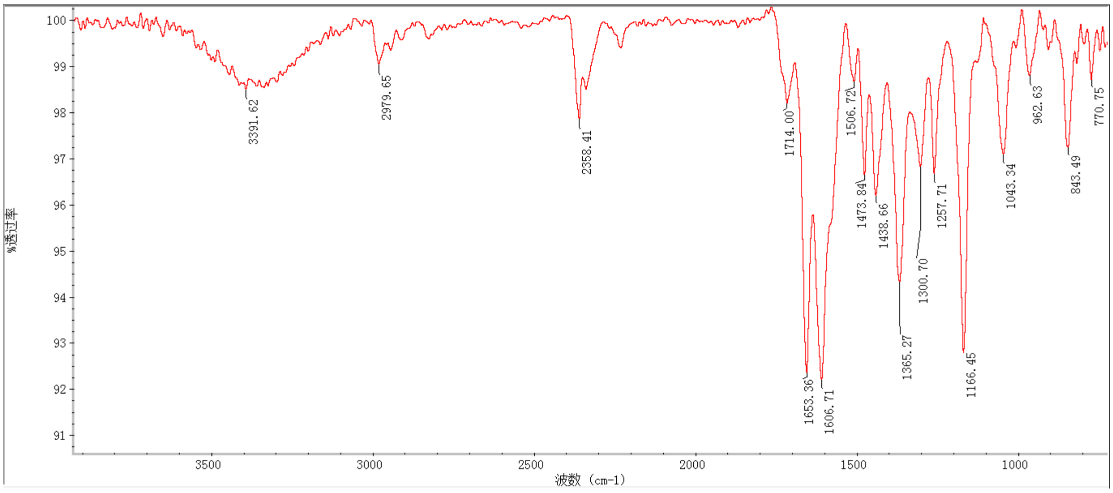

Figure S9. IR spectrum of **1**.

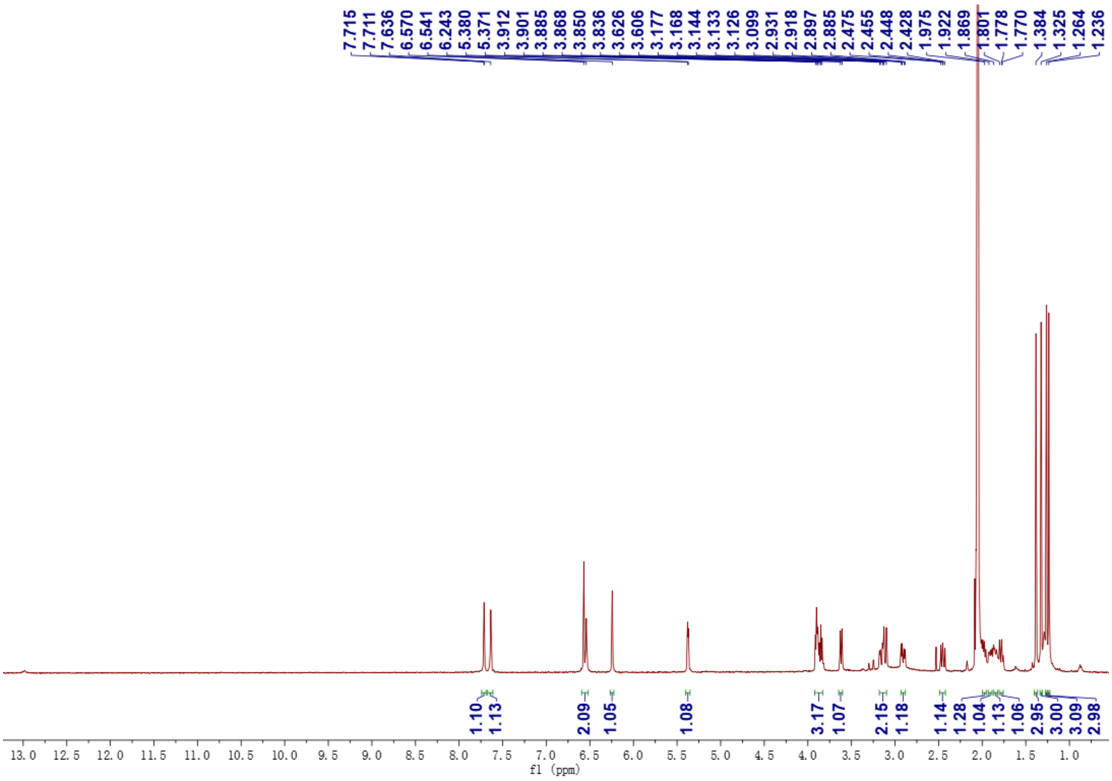

Figure S10. ^{1}H NMR (500 MHz) spectrum of **2** in Acetone-d_6.

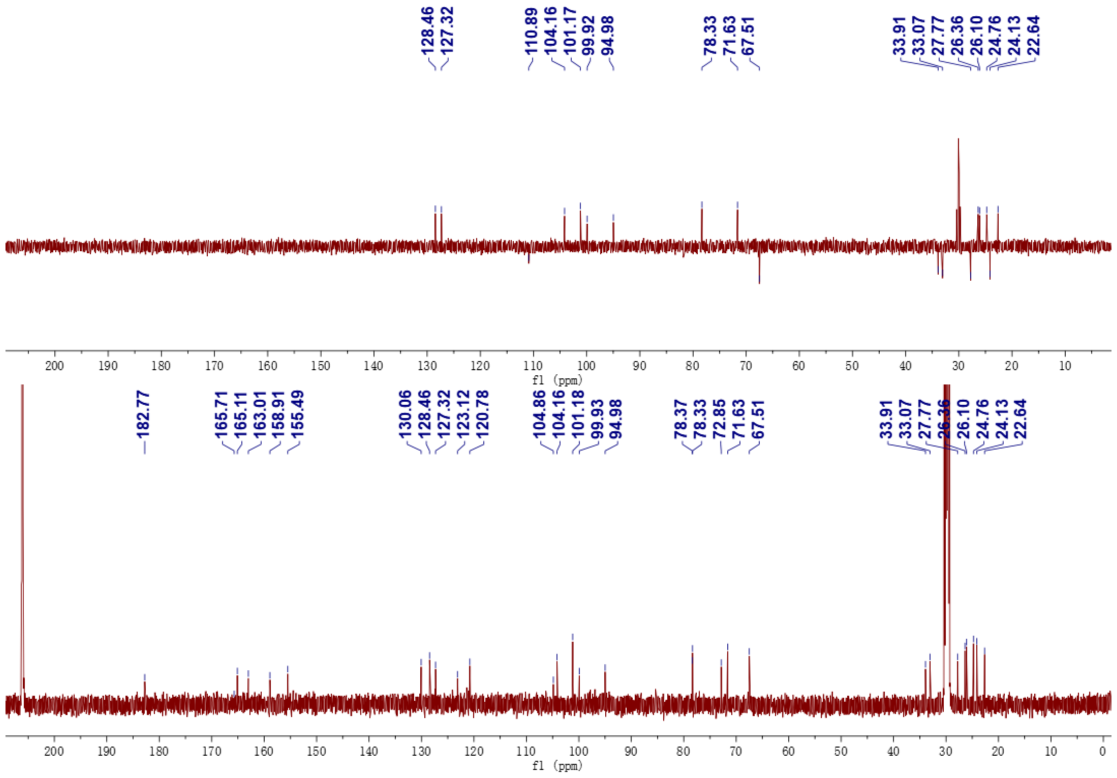

Figure S11. DEPT and ^{13}C NMR (125 MHz) spectra of **2** in Acetone-d_6.

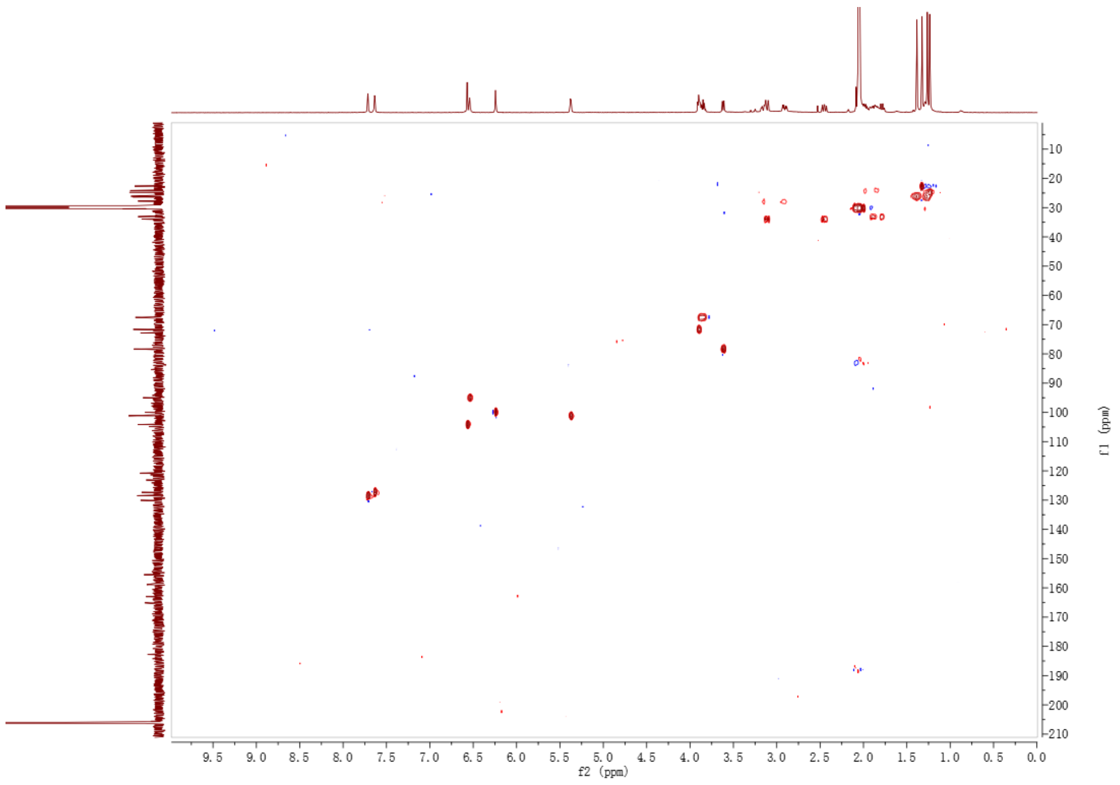

Figure S12. HSQC NMR spectrum of **2** in Acetone-d_6.

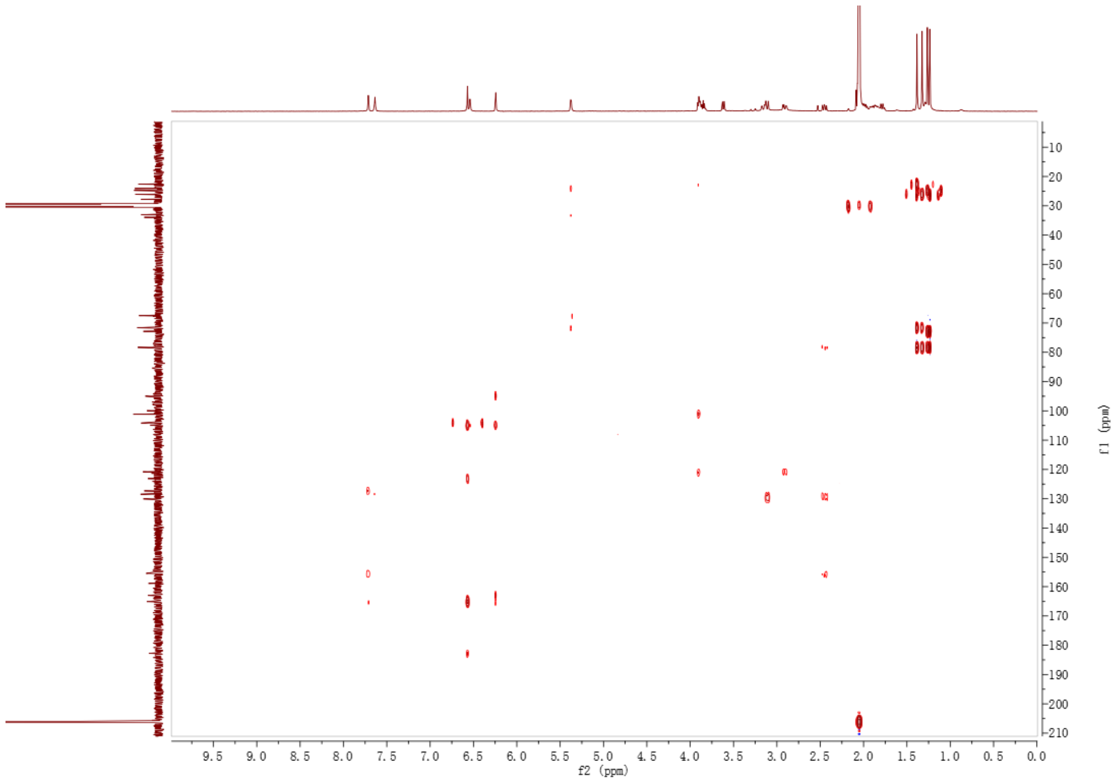

Figure S13. HMBC NMR spectrum of **2** in Acetone-d_6.

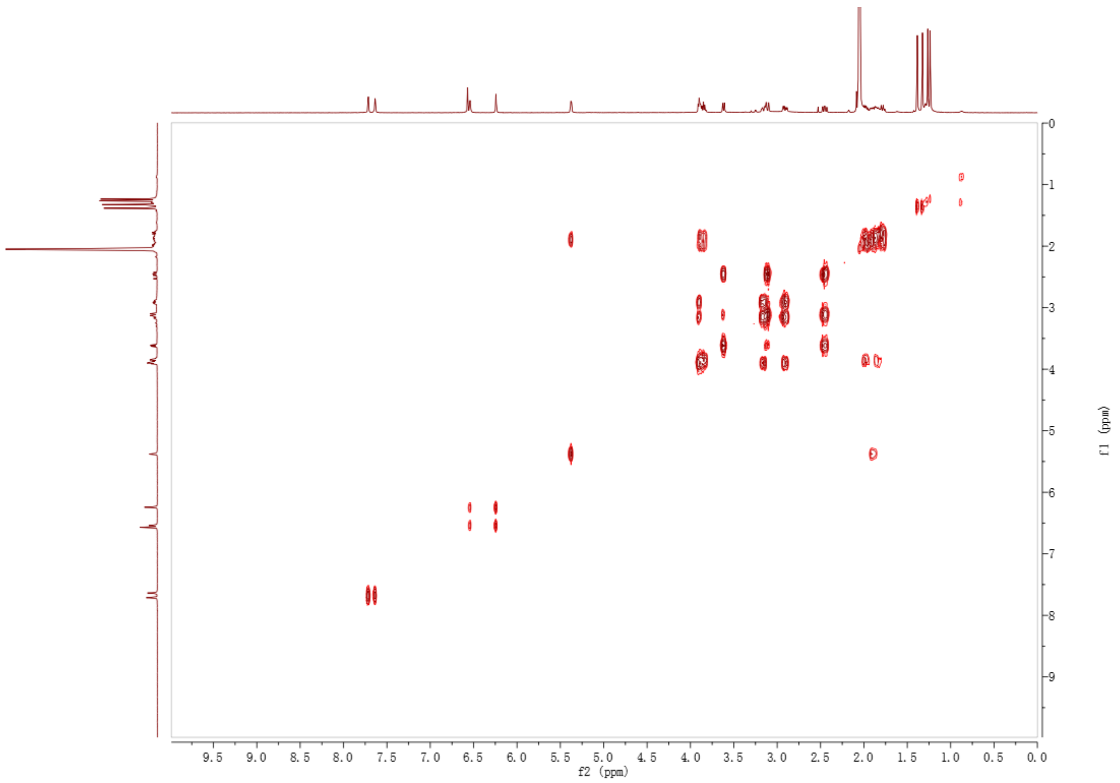

Figure S14. ^{1}H-^{1}H COSY NMR spectrum of **2** in Acetone-d_6.

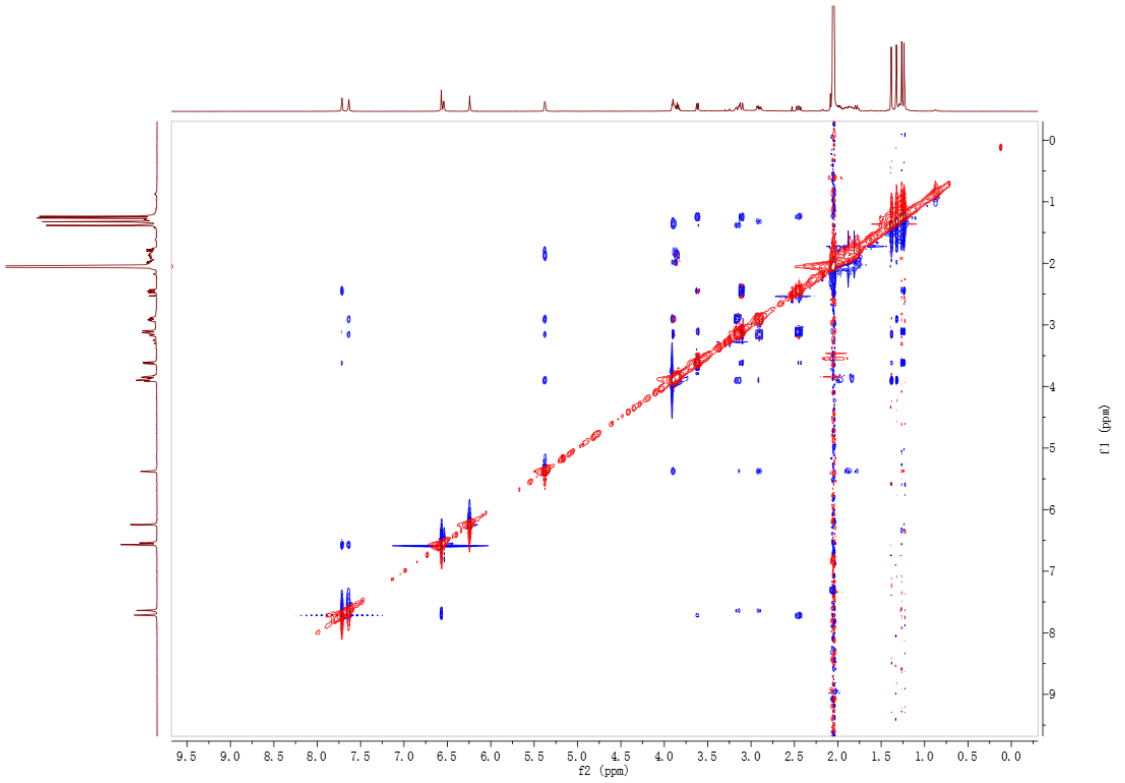

Figure S15. NOESY NMR spectrum of **2** in Acetone-d_6.

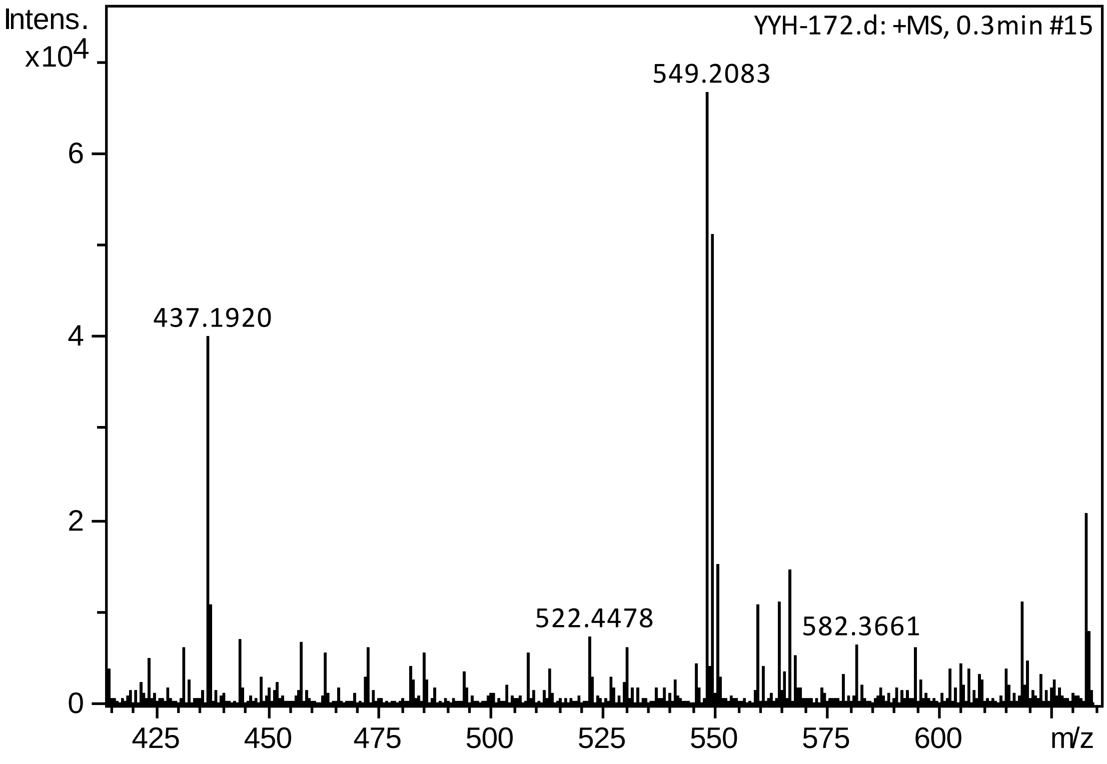

Figure S16. HRESIMS spectrum of **2**.

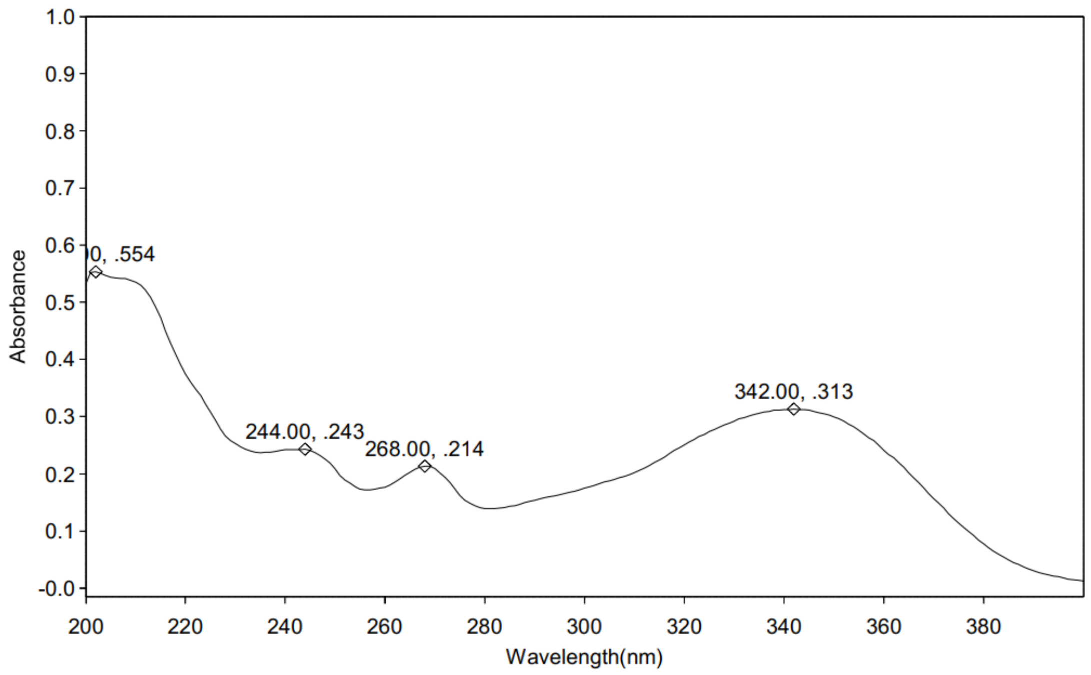

Figure S17. UV spectrum of **2**.

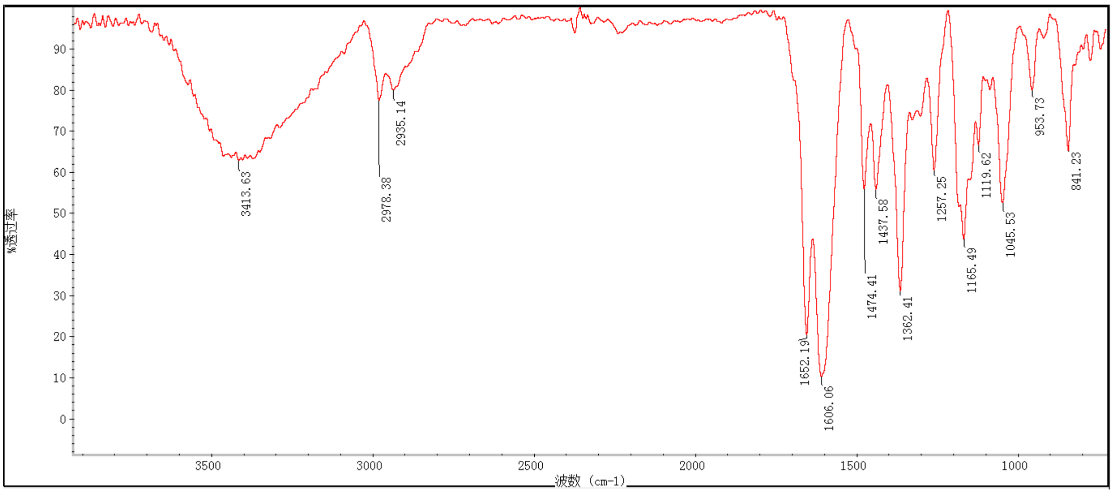

Figure S18. IR spectrum of **2**.

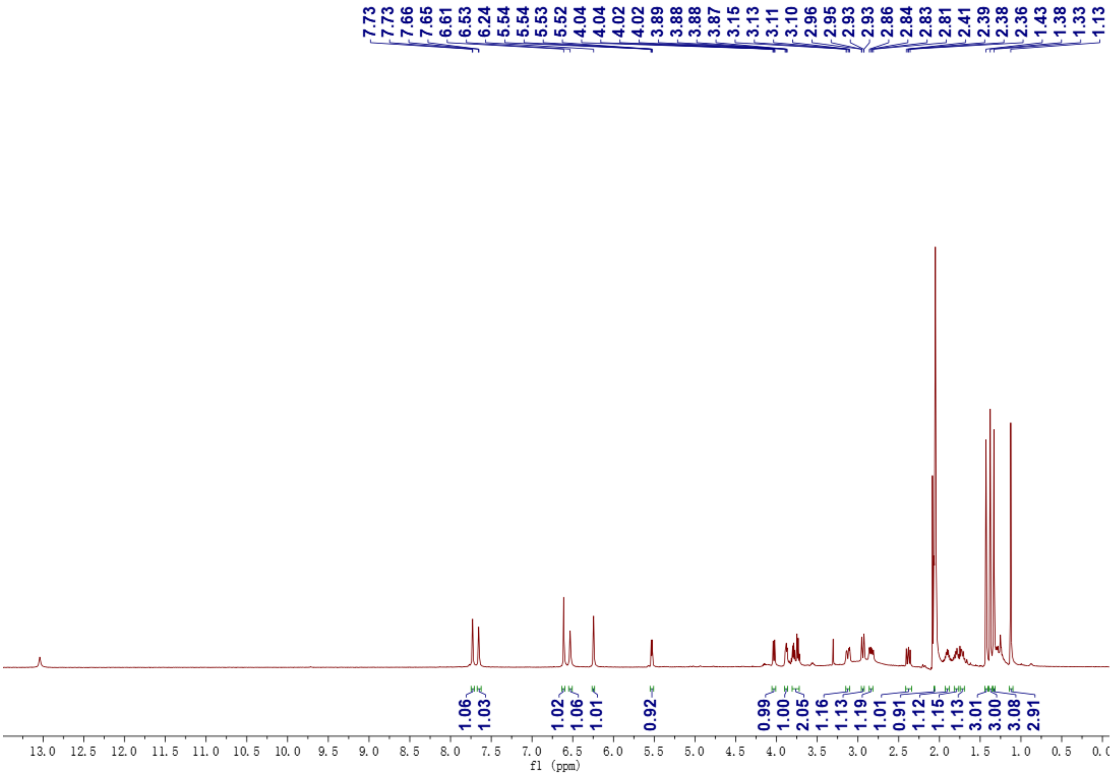

Figure S19. ^{1}H NMR (500 MHz) spectrum of **3** in Acetone-d_6.

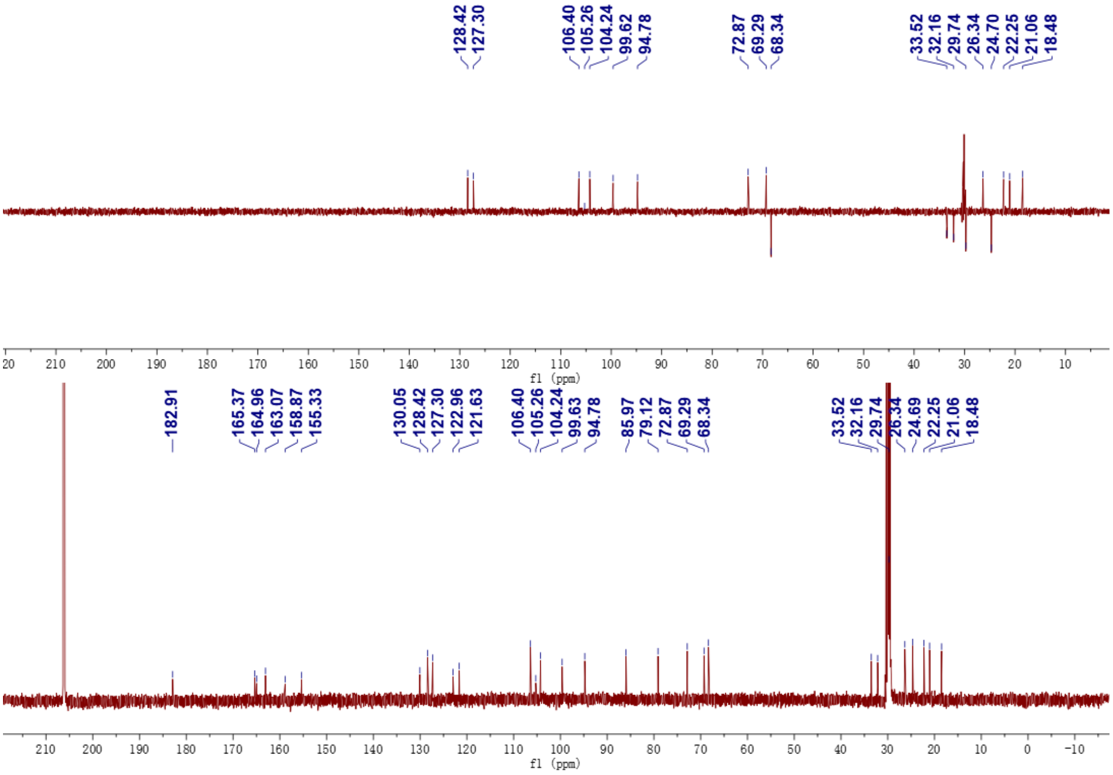

Figure S20. DEPT and ^{13}C NMR (125 MHz) spectra of **3** in Acetone-d_6.

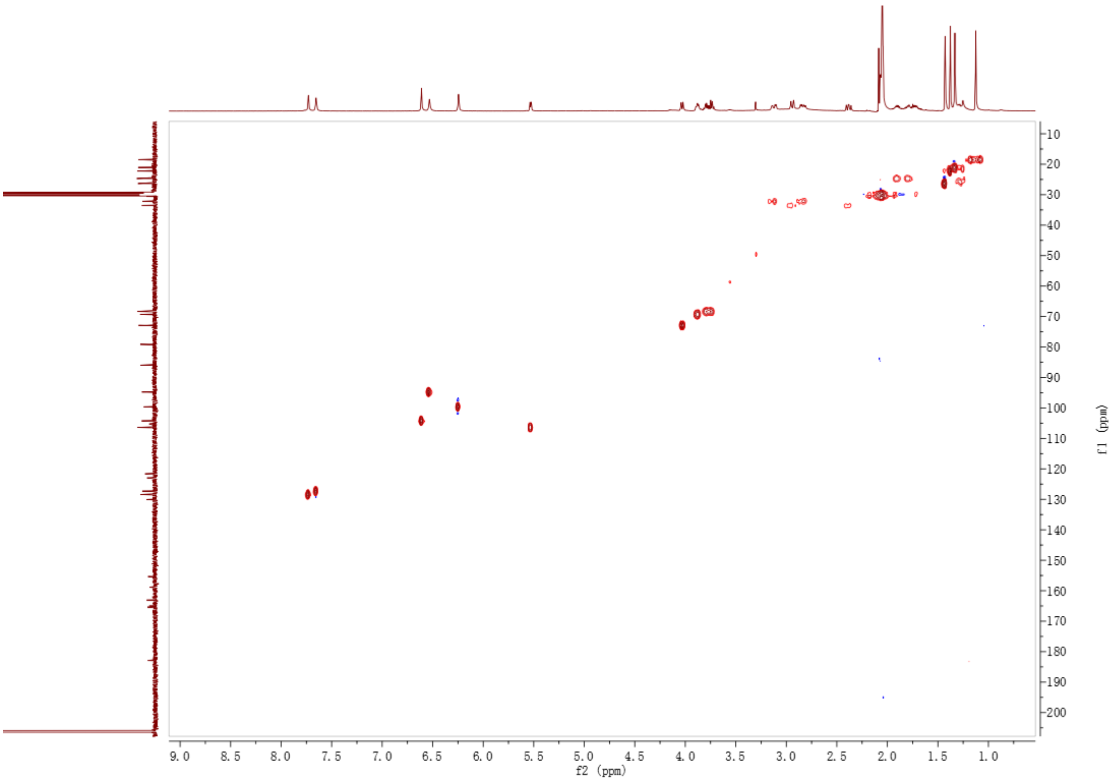

Figure S21. HSQC NMR spectrum of **3** in Acetone-d_6.

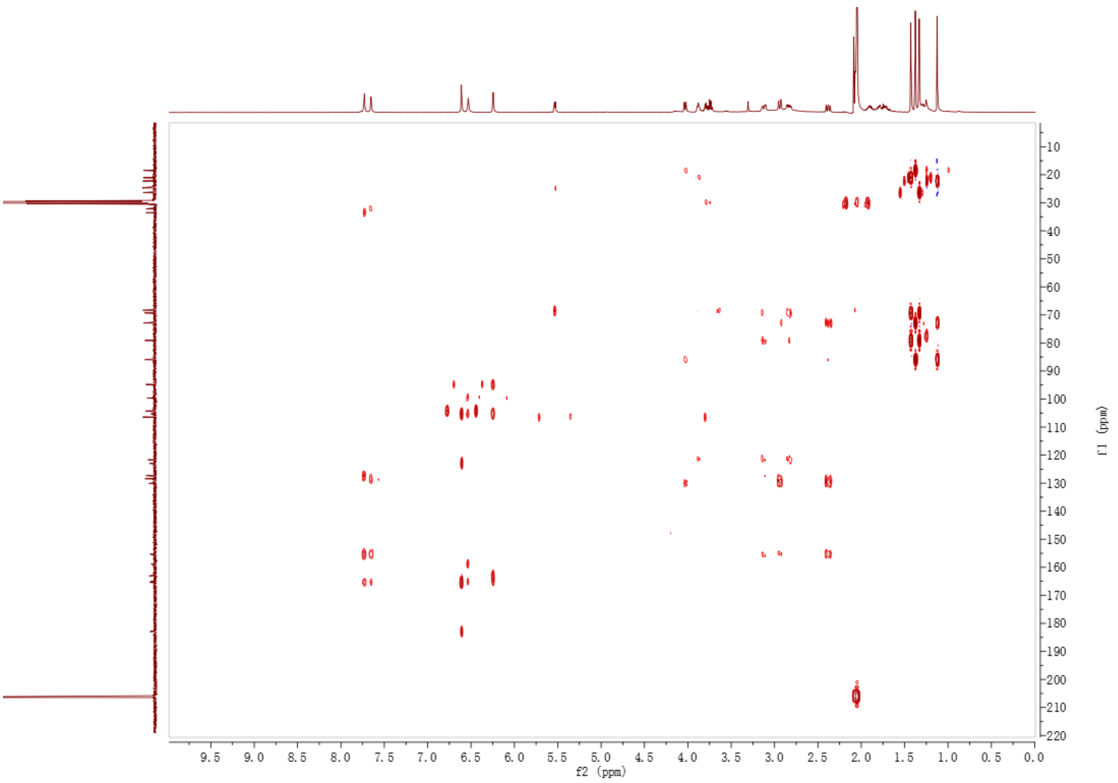

Figure S22. HMBC NMR spectrum of **3** in Acetone-d_6.

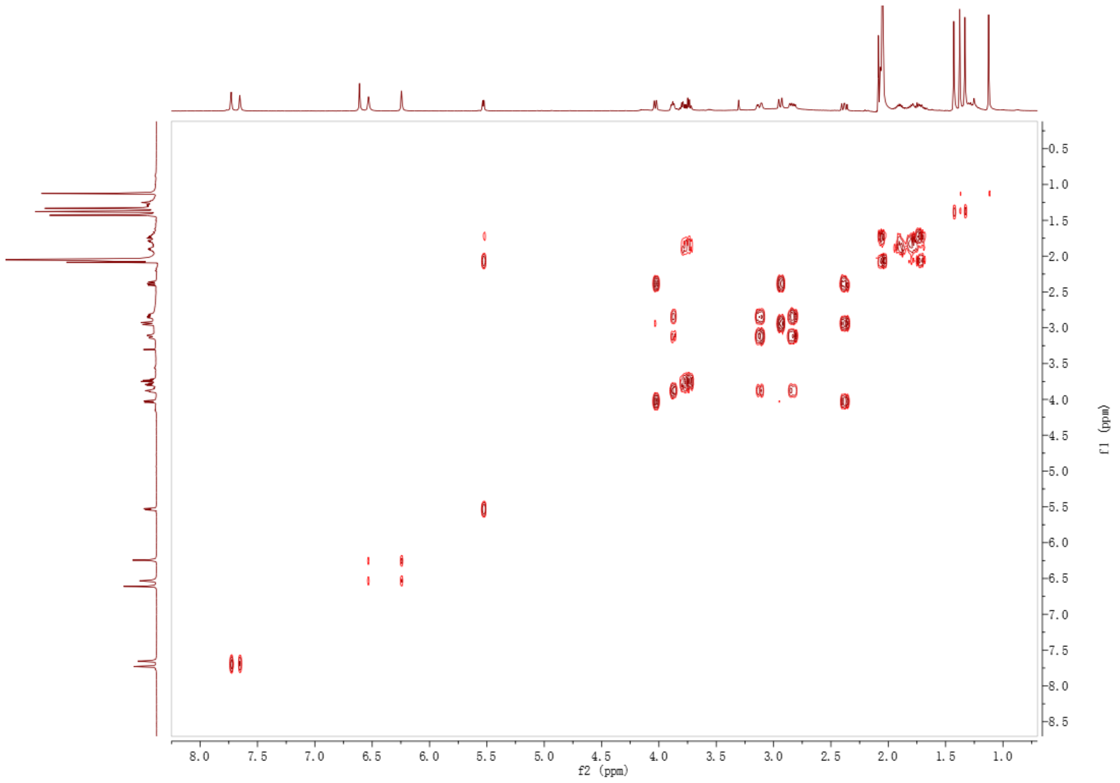

Figure S23. ^1H-^1H COSY NMR spectrum of **3** in Acetone-d_6.

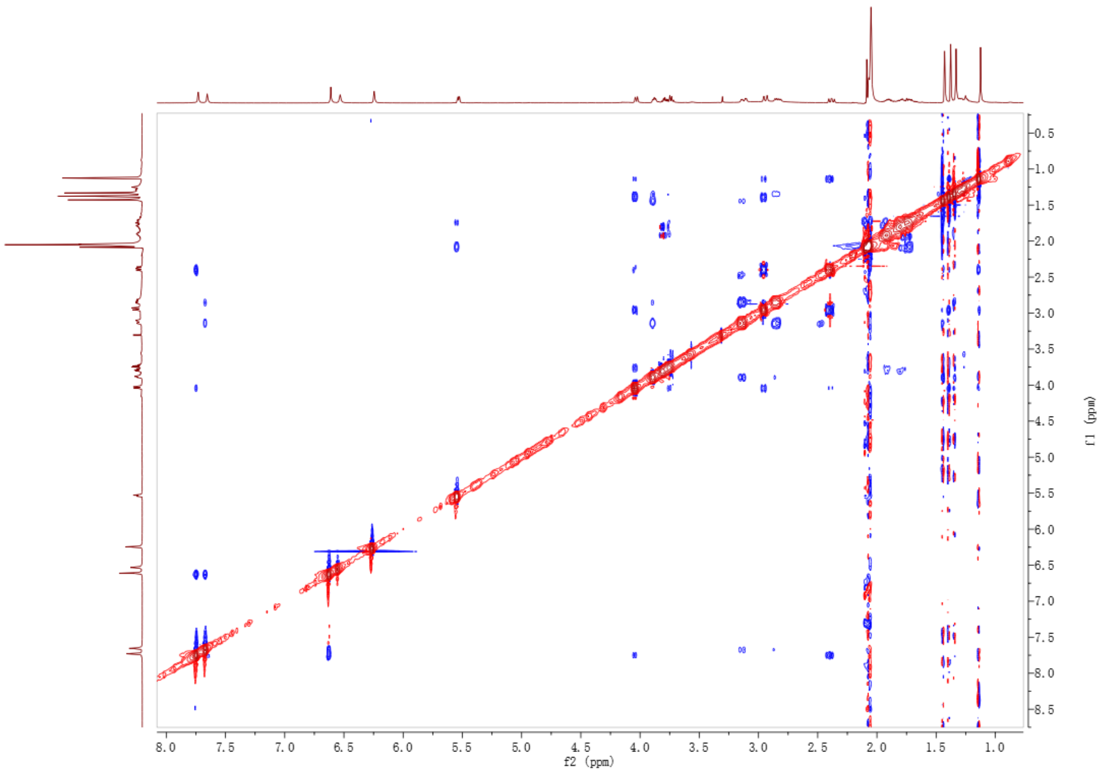

Figure S24. NOESY NMR spectrum of **3** in Acetone-d_6.

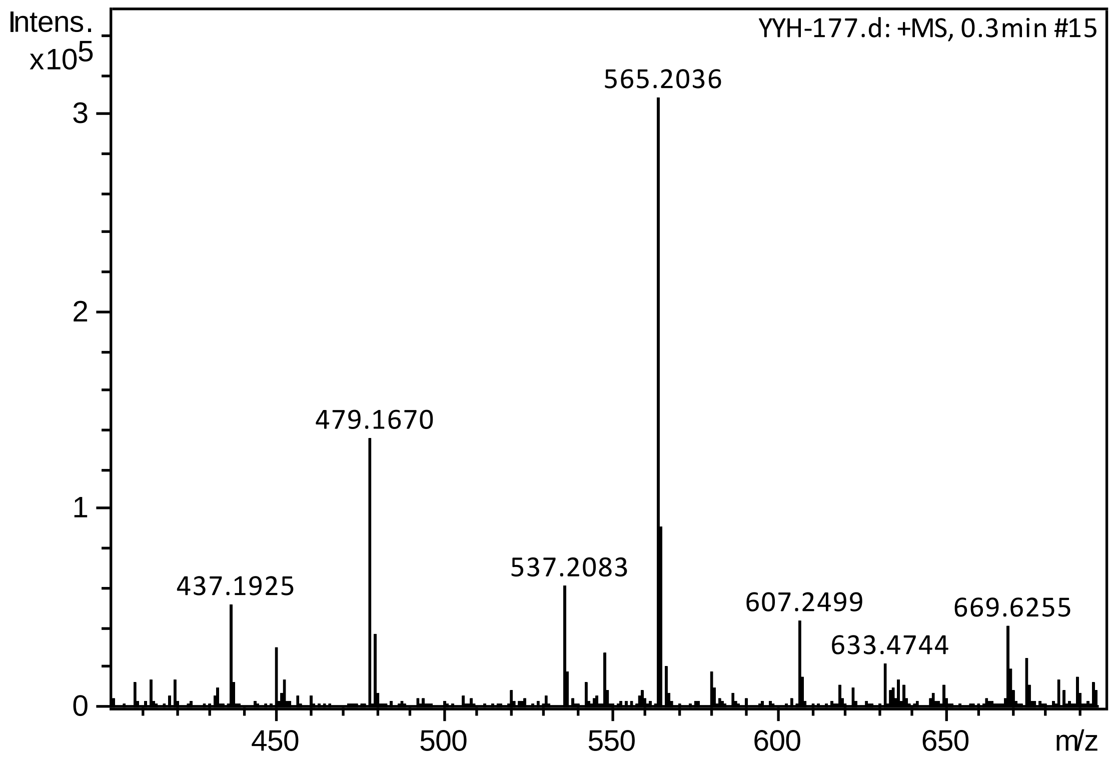

Figure S25. HRESIMS spectrum of **3**.

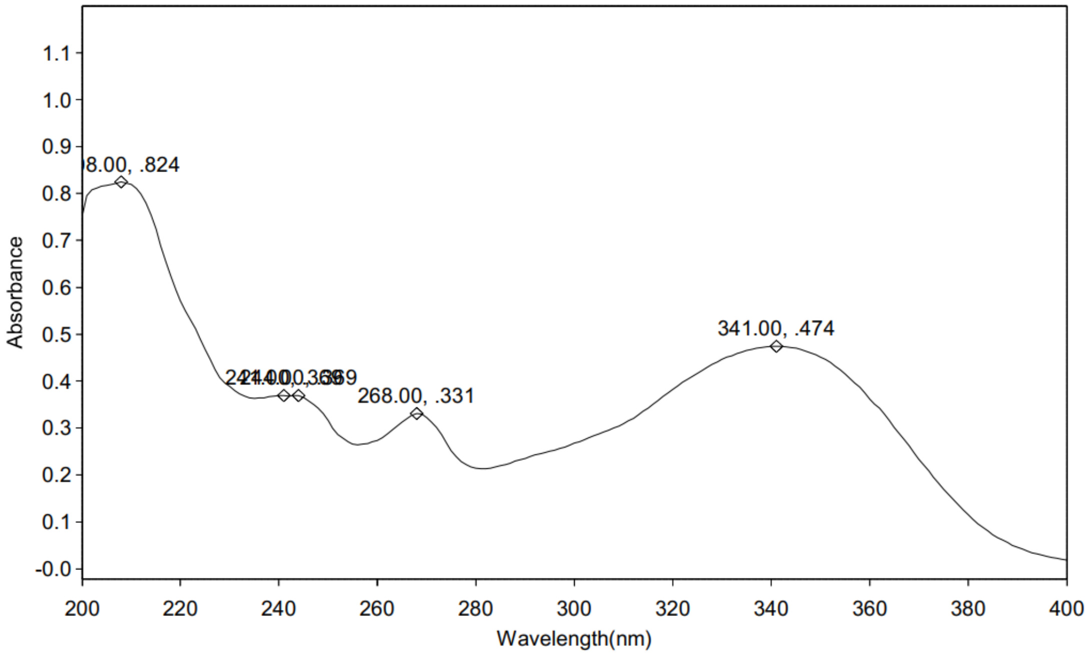

Figure S26. UV spectrum of **3**.

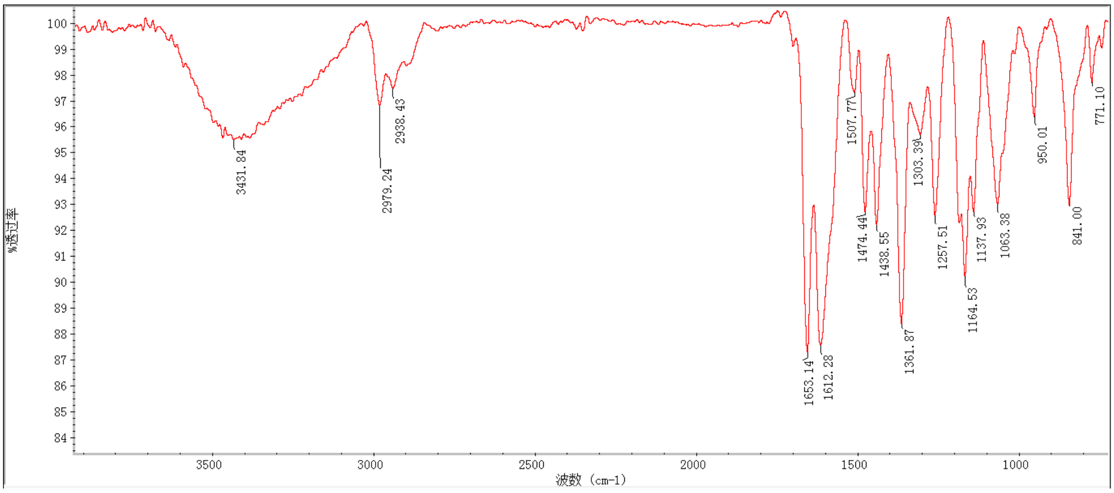

Figure S27. IR spectrum of **3**.

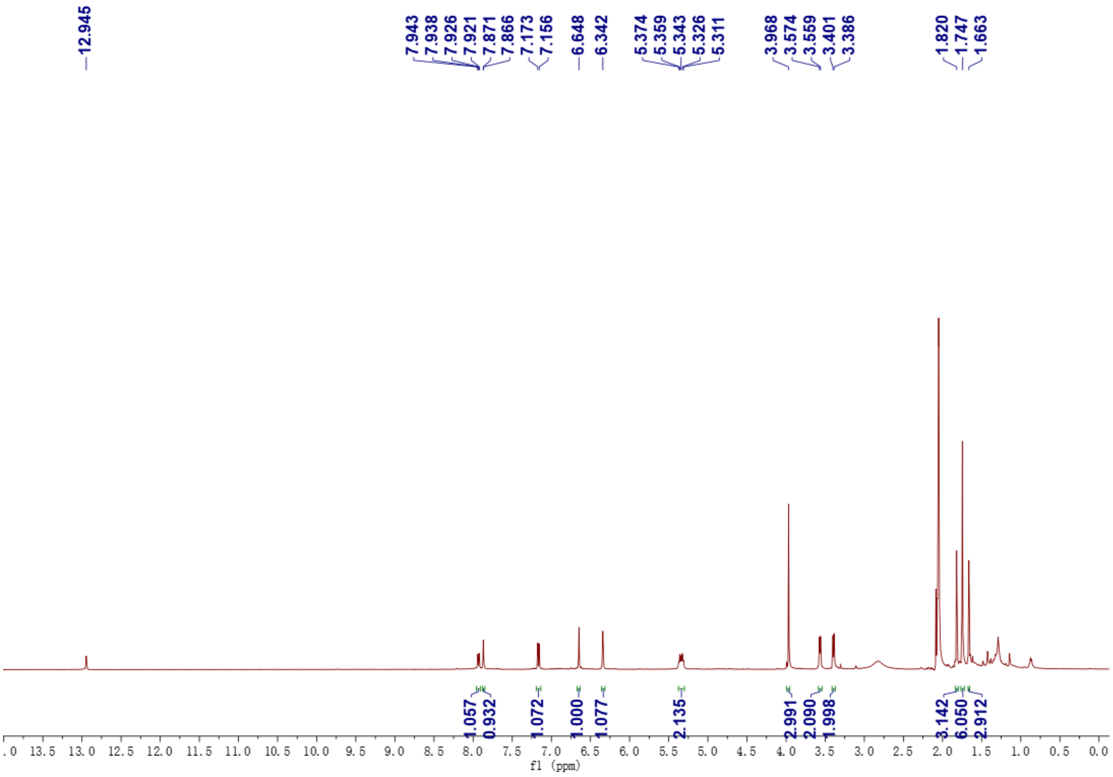

Figure S28. ^{1}H NMR (500 MHz) spectrum of **4** in Acetone-d_6.

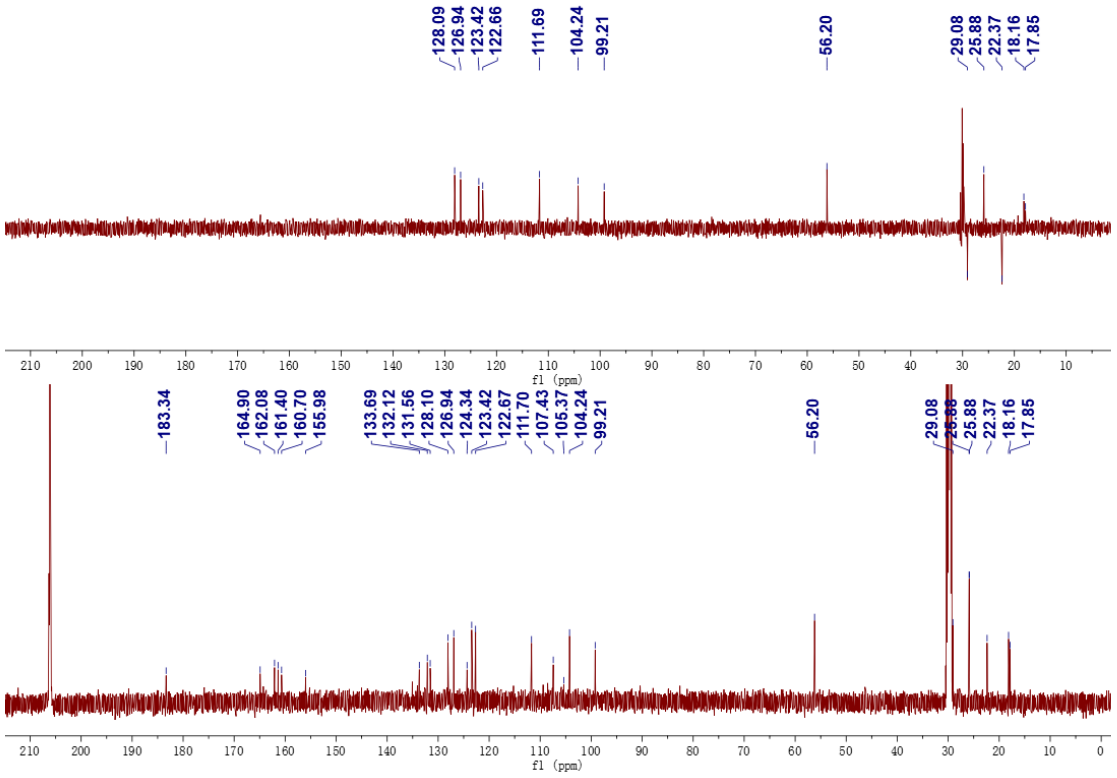

Figure S29. DEPT and ^{13}C NMR (125 MHz) spectra of **4** in Acetone-d_6.

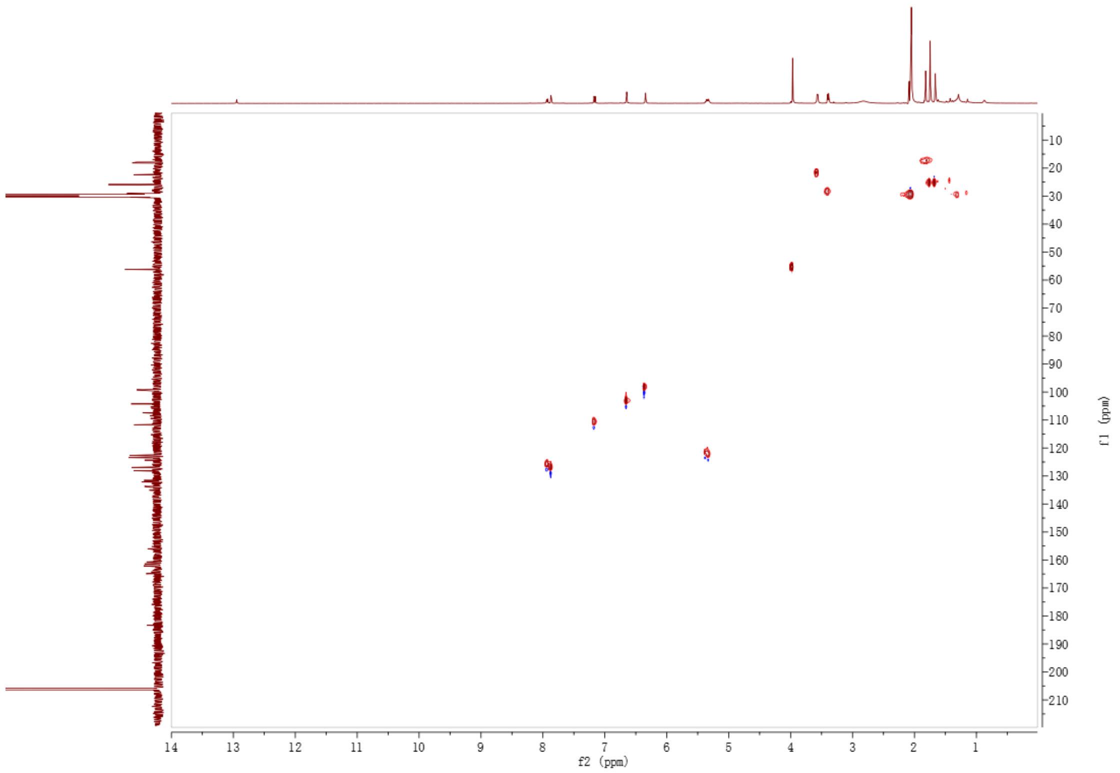

Figure S30. HSQC NMR spectrum of **4** in Acetone-d_6.

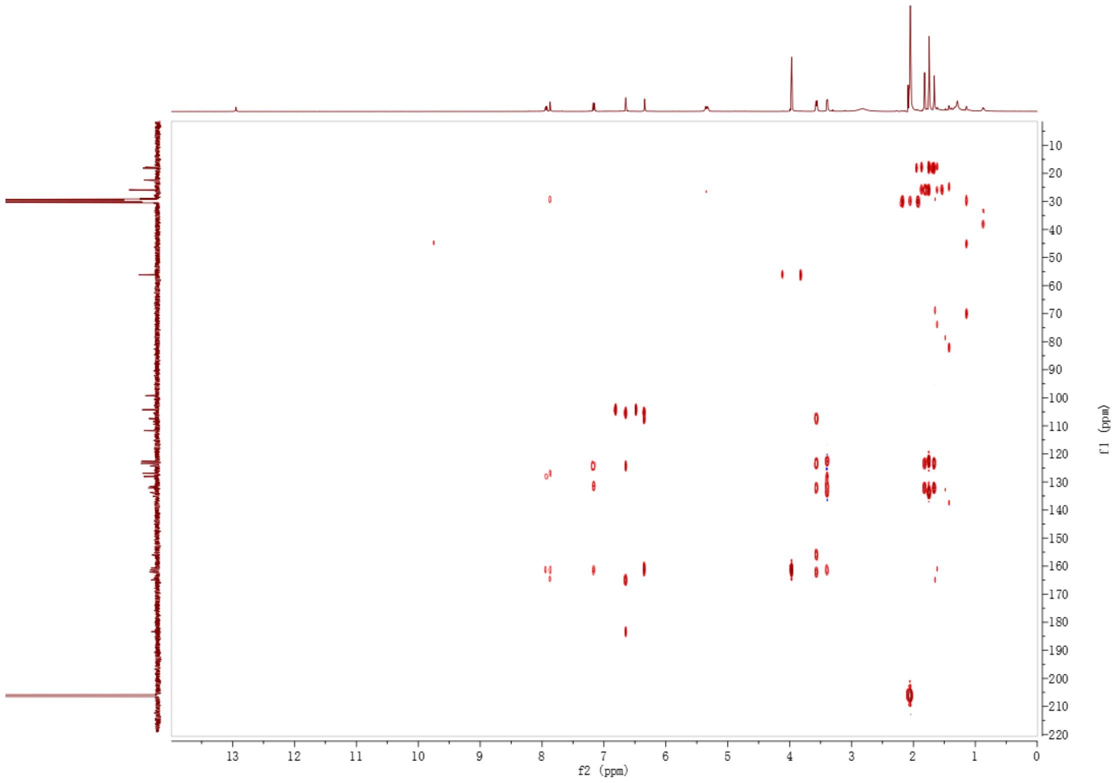

Figure S31. HMBC NMR spectrum of **4** in Acetone-d_6.

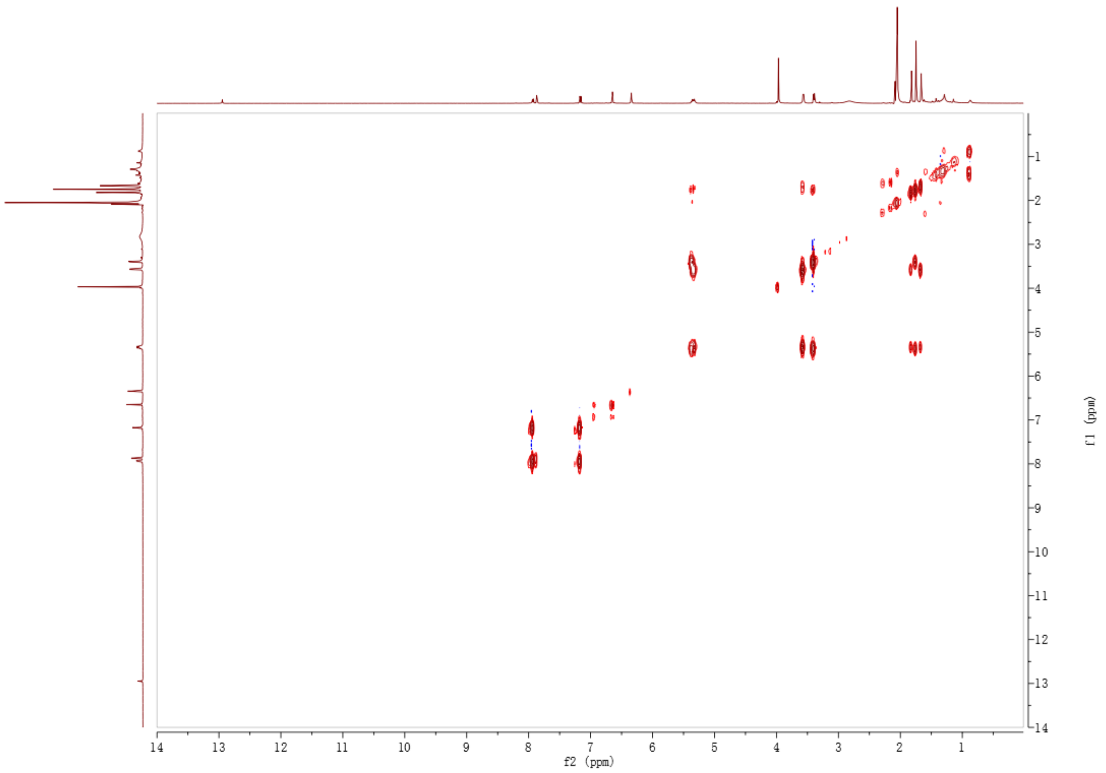

Figure S32. ^{1}H-^{1}H COSY NMR spectrum of **4** in Acetone-d_6.

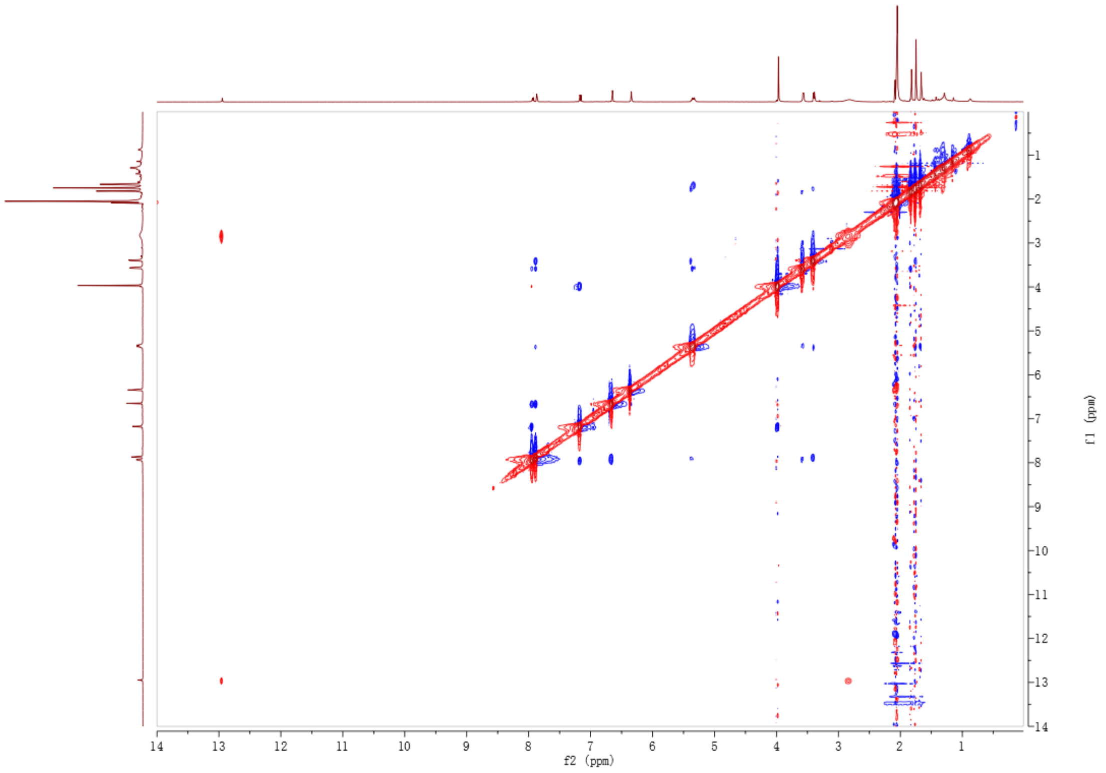

Figure S33. NOESY NMR spectrum of **4** in Acetone-d_6.

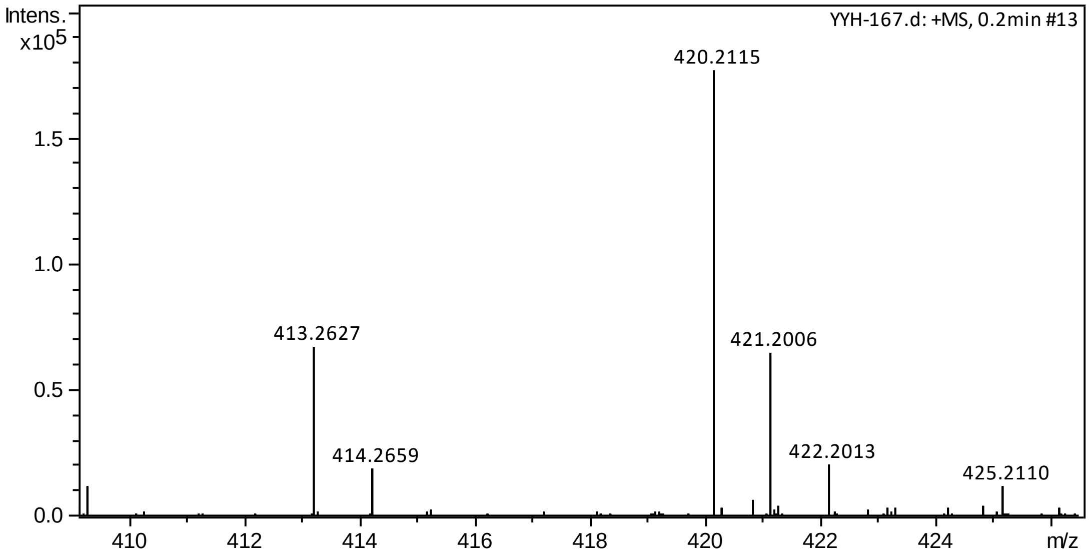

Figure S34. HRESIMS spectrum of **4**.

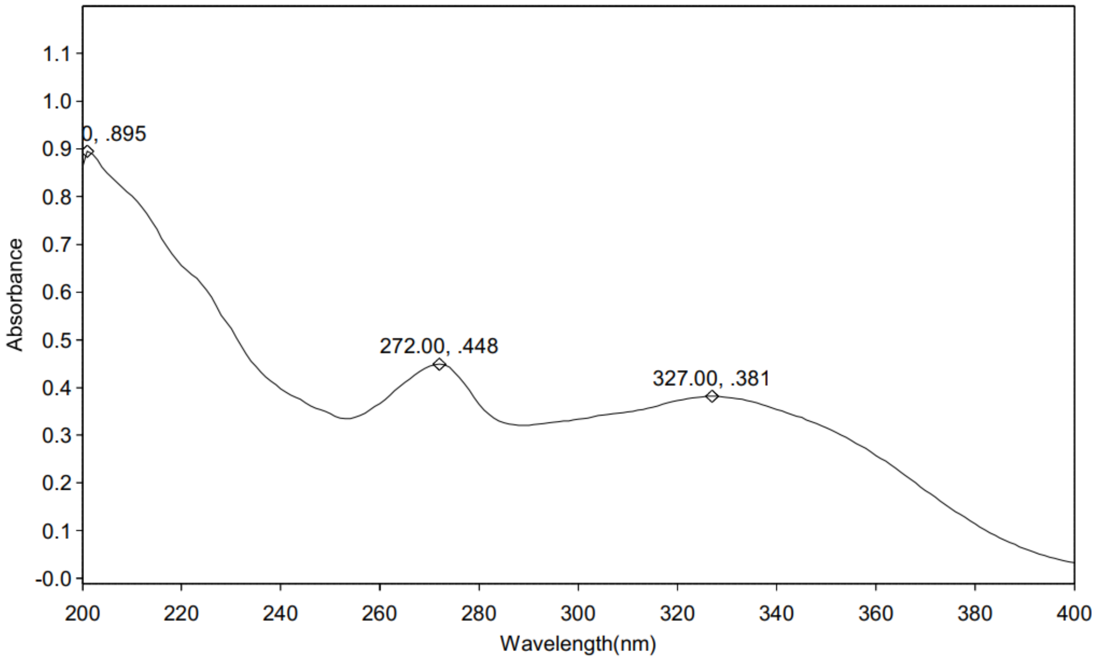

Figure S35. UV spectrum of **4**.

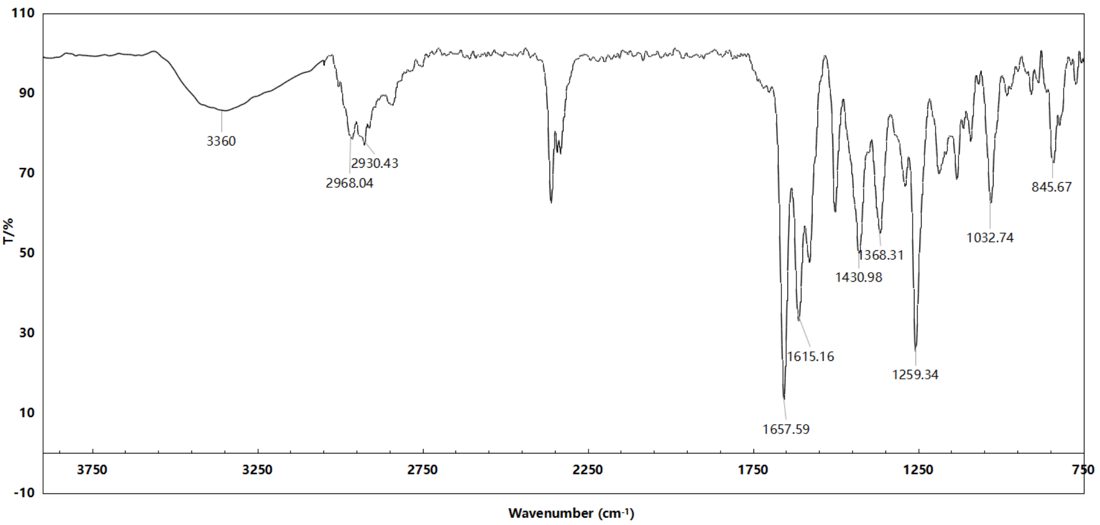

Figure S36. IR spectrum of **4**.
